# Effects of hatching system on chick quality, welfare and health of young breeder flock offspring

**DOI:** 10.1016/j.psj.2022.102448

**Published:** 2022-12-21

**Authors:** Roos Molenaar, Norbert Stockhofe-Zurwieden, Mona F. Giersberg, T. Bas Rodenburg, Bas Kemp, Henry van den Brand, Ingrid C. de Jong

**Affiliations:** ⁎ Adaptation Physiology Group, Wageningen University and Research, Wageningen, The Netherlands; † Animals in Science and Society, Faculty of Veterinary Medicine, Utrecht University, Utrecht, The Netherlands; ‡ Wageningen Livestock Research, Wageningen University and Research, Wageningen, The Netherlands; § Wageningen Bio-Veterinary Research, Wageningen University and Research, Lelystad, The Netherlands

**Keywords:** hatching system, broiler chicken, eggshell temperature, welfare, resilience

## Abstract

Alternative hatching systems have been developed for broiler chickens to provide immediately feed and water after hatch and reduce the number or severity of early life stressors. Besides beneficial effects of these alternative hatching systems on chick quality and performance, broiler health and welfare may be positively affected as well. Especially offspring from young broiler breeder flocks may benefit, as they have been shown to be more sensitive to preturbations than offspring from older breeder flocks. This study evaluated effects of hatching systems on chick quality, health and welfare of young breeder flock offspring, using 3 different hatching systems: conventional hatchery-hatched (**HH**), hatchery-fed (**HF**), and on-farm hatching (**OH**). A total of 24 pens were used in a completely randomized block design, with 8 pens per hatching system and 30 chickens per pen. Chick quality at hatch and performance until 35 d of age was improved in the HF and OH compared to HH treatment, but only minor effects were found on the welfare indicators: footpad dermatitis, hock burn, cleanliness, skin lesion and gait score. No effect was observed on the dynamics of a humoral immune response after NCD vaccination, given at d 0 and 14 of age, as no differences between NCD titers were found at d 18. Animals were vaccinated with a live attenuated infectious bronchitis vaccine virus (**IBV**) at d 28 to address treatment related differences to disease resilience. The expressions of inflammation and epithelial integrity related genes in the trachea and histo-pathological changes in the trachea were examined at 3 d after vaccine administration. No differences between treatment groups were observed. Although beneficial effects of HF and OH systems were found for young breeder flock offspring on chick quality at hatch and body weight posthatch, only one effect of alternative hatching systems on welfare and health indicators were found. No effect of hatching system on humoral immune response or disease resilience was found.

## INTRODUCTION

Chickens that hatch in conventional hatching systems can have a delay in feed and water access up to 72 h because of hatch and/or pull time differences, handling and processing procedures and transportation to the rearing farm (Noy and Sklan, 1999; Decuypere et al., 2001). Moreover, chickens in conventional hatching systems hatch in darkness and are exposed to noise and dust (Archer and Mench, 2014; de Gouw et al., 2017; Hedlund et al., 2019). These aspects may negatively affect posthatch survival and performance and impair chicken welfare (de Gouw et al., 2017; Archer, 2018). To address these risks, alternative hatching systems have been developed for broiler chickens, providing feed and water immediately after hatch. Also several environmental factors and handling procedures differ between these alternative hatching systems and conventional hatchery systems, possibly contributing to day-old chick quality, posthatch performance and health and welfare. First, chicken embryos are exposed to light during their hatching process in alternative hatching systems, which may reduce fear response and stress susceptibility (Archer and Mench, 2013, Archer and Mench, 2017). Second, noise level is lower in especially on-farm hatching systems, which can contribute to synchronize the hatching process and shorten the hatch window within a batch of eggs (Van de Ven et al., 2010). Third, air velocity and air temperature is often lower in alternative hatching systems that provide feed and water immediately after hatch (Van de Ven, 2012). This may affect the temperature that the embryo experiences and consequently influence embryonic development, day-old chick quality as well as post hatch performance and resilience (Leksrisompong et al., 2007; Maatjens et al., 2016; Wijnen et al., 2020). Finally, processing and handling of chickens is less or even nihil in alternative hatching systems, and this reduces the number of early-life stressors and may positively affect behavior, welfare, and performance of chickens in later life (Ericsson et al., 2016; Giersberg et al., 2021; Hedlund and Jensen, 2021).

Currently, 2 types of alternative hatching systems providing feed and water immediately after hatch are available on the market. Chickens either hatch at the hatchery and are immediately provided with feed and water but still need to be processed and/or transported to the broiler farm (Souza da Silva et al., 2021), or fertile eggs at d 18 of incubation are transported to and hatch at the broiler farm (de Jong et al., 2019). Although there are extensive studies about effects of providing feed and water immediately after hatch on later life performance (Noy et al., 2001; Juul-Madsen et al., 2004; El-Husseiny et al., 2008; de Jong et al., 2017; Ivarsson et al., 2022), there are only a few comparisons with alternative hatching systems that also comprise welfare and health aspects (de Jong et al., 2019; Souza da Silva et al., 2021; Jessen et al., 2021a; Jessen et al., 2021b). The studies that were performed showed that alternative hatchery systems resulted in less footpad dermatitis in later life of broiler chickens thereby directing to an improved of welfare and health (De Jong et al., 2018; de Jong et al., 2020, Giersberg et al., 2021). Effects of alternative hatching systems on health aspect such as immune response and disease resilience has been poorly investigated. However, studies about early feeding indicated changes within immune system development and improvement in resilience indicators in early fed compared to delayed fed broiler chickens, suggesting an increased ability to maintain a relatively undepressed performance in the face of an infection (Albers et al., 1987; Mulder and Rashidi, 2017). Hollemans et al. (2021) showed that up to an age of 7 d maturation of the humoral immune system was enhanced in early fed broilers compared to 72 h delayed fed broiler chickens. Wijnen et al. (2021) found a tendency for a lower mortality after a necrotic enteritis infection in broiler chicks that were immediately fed compared to chickens with a 51 to 54 h delay in feed and water provision.

Finally, chickens from young breeder flock offspring (<35 wk of age) that hatch in alternative hatching systems have shown more often an improved performance until slaughter age compared to chickens from prime and old breeder flock offspring (de Jong et al., 2019, 2020; Jessen et al., 2021b). This may be related to the smaller size and body composition of chickens of young compared to older breeder flocks at hatch (McNaughton et al., 1978; Nangsuay et al., 2013) possibly in combination with a different sensitivity to early life stressors (Peixoto et al., 2021).

The aim of the current study was therefore to evaluate effects of two alternative hatching systems (hatchery-feeding and on-farm hatching) compared to conventional hatching systems with respect to chick quality, welfare and health of a young breeder flock. We hypothesized that alternative hatching systems due to the added effects of early feed and water provision and the lower number of early stressors would result in better health and welfare by an improved immune system development and disease resilience. To study the effect of treatments on the competence of the humoral immune response, blood titers after a live attenuated Newcastle Disease (**NCD**) vaccination was assessed. To study differences in disease resilience, the susceptibility to develop tracheal inflammation after infection with a live attenuated Infectious Bronchitis (**IB**) vaccine virus was assessed by trachea lesion scoring and expression of genes related to epithelial integrity and inflammatory responses.

## MATERIAL AND METHODS

### Experimental Design

In the current study, 3 treatment groups were included: the conventional hatchery-hatched system (**HH**), and two alternative systems: hatchery-fed system (**HF**) or on-farm hatching system (**OH**). A total of 24 pens was used in a completely randomized block design, with 8 pens per hatching system and 30 broiler chickens per pen. The experimental protocol was approved by the Governmental Commission on Animal Experiments, The Hague, The Netherlands, approval number 2019.D-0002.002.

### Incubation Period and Processing Procedures

In total, 1,215 first grade hatching eggs of a Ross 308 breeder flock of 27 wk were set in a HatchTech Microclimer (capacity of 57,600 eggs, HatchTech, Veenendaal, The Netherlands) at a commercial hatchery in the Netherlands (Lagerwey, Lunteren, The Netherlands). Eggs were placed in 9 egg trays containing 135 eggs each and trays were placed in the middle of a full setter trolley. The rest of the incubator was loaded with trolleys containing eggs from other breeder flocks, which were not part of the study. Eggs were heated from storage room temperature (18°C) to the desired incubation temperature in 23 h. Incubation temperature was set at 38.3°C at d 0 of incubation (start of incubation process) and decreased gradually to 37.2°C at d 18 of incubation. CO_2_ levels were maintained below 35 ppm throughout the incubation process and relative humidity was maintained between 30 and 75% at d 0 of incubation and gradually decreased to a range between 20 and 40% at d 18 of incubation. Eggs were turned hourly over 90° until d 18 of incubation. Weight per tray was measured at set and individual egg weight was calculated (average 55 g). All egg trays were weighed again at d 18.5 of incubation and weight loss per egg tray until d 18.5 of incubation was calculated (average 11.2%). At d 18.5 of incubation, all eggs were candled, and fertility and embryonic mortality were calculated (average 97.4% and 4.2%, respectively). After candling, 108 fertile eggs from each tray were randomly allocated to one of the 3 hatching systems, resulting in 9 repetitions of 36 eggs per treatment.

In the hatchery-hatched (**HH)** treatment, eggs were transferred to 9 hatching baskets and set within a HatchTech Picoclimer (capacity of 4,800 eggs, HatchTech, Veenendaal, The Netherlands) in the same hatchery as for the setter phase. Temperature was set at 36.4°C and decreased to 35.0°C at d 21 of incubation. CO_2_ levels were maintained below 20 ppm and relative humidity was maintained between 50% and 65% until processing at approximately 510 h after the start of incubation. Air speed within the hatcher was approximately 1.5 m/s. Unhatched eggs were collected per basket and hatched chickens were transported per basket over the processing belts where hatchery staff removed the second-grade chickens, after which the chickens went through the chick counter machine (Viscon, ‘s Gravendeel, The Netherlands). Second grade chickens were categorized when chickens were small and/or unable to stand or showed deformities such as unabsorbed yolk or open navel area, crossed beak, exposed brains, 4 legs (van de Ven et al., 2012; Wijnen et al., 2020). Chickens were feather sexed and 15 males and 15 females from each hatching basket were randomly selected and allocated to a pen at the research facility. Nine baskets (595 × 397 ×166 mm) with 30 chickens each were transported in darkness at 30°C by a climate-controlled van for 30 min to the research facility of Wageningen University and Research (Wageningen, The Netherlands). No feed or water was provided to the hatchery-hatched chickens until placement in the pens at the research facility, which was approximately 25 to 30 h after the chickens hatched.

With the hatchery-fed (**HF**) treatment, eggs were transferred at d 18.5 of incubation to HatchCare cradles (673 × 580 × 166 mm) and transported for 2 h at 30°C in a climate-controlled van to another hatchery within the Netherlands (Probroed, Langenboom, The Netherlands) and set within a HatchCare system (HatchTech B.V., Veenendaal, The Netherlands), where feed and water was available immediately after hatching. Further details about the design of the HatchCare system can be found in Souza da Silva et al. (2021). Temperature was set at 37.2°C and decreased to 35.6°C at d 21 of incubation. CO_2_ levels were maintained below 25 ppm and relative humidity was maintained between 25% and 50% until processing at approximately 516 h of incubation. Air speed within the HatchCare system was approximately 0.5 m/s. Chickens remained in their cradle during processing and non-hatched eggs and second-grade chickens were taken out manually by hatchery staff. The same procedure for feather sexing was applied as described above for the HH treatment. Nine HatchCare cradles with 30 chickens each were transported in darkness at 30°C by a climate-controlled van for 45 min to the same research facility as described above for the HH treatment. The remaining of the prestarter diet that was provided in the HatchCare system stayed in the cradles during transport (prestarter diet: 2,900 kcal ME/kg; CP 21%, CF 6%).

In the on-farm hatching (**OH**) treatment, eggs were candled and transferred to egg trays at d 18.5 of incubation and transported to the same research facility as described for the HH treatment and 36 fertile eggs were placed per pen in a small prototype of the X-Treck system (Vencomatic, Eersel, The Netherlands). The prototype was placed in the middle of the pen and consisted of a honeycomb structured egg tray for 88 eggs which was placed on a wooden frame at 22 cm above the floor. Halfway between the floor and the egg tray, there was a small static conveyor belt present containing a thin layer of fresh wood shavings. After hatching, chickens fell on the conveyor belt, dried and then fell on the floor of the pen. Upon arrival of the 18 d old fertile hatching eggs, floor temperature was maintained at 28°C and room temperature was maintained at 34°C. Air speed was <0.2 m/s. Eggshell temperature of 4 eggs per pen were measured 4 times a day with an ear thermometer (Braun, The Netherlands) and room temperature was adjusted to maintain an eggshell temperature between 37°C and 38°C until d 19 of incubation. When the first chickens started to hatch, feed and water was provided in the pen. A crumbled diet was provided (find details in the next paragraph). Chickens were feather sexed at 510 h of incubation (= d 0 of the growout period) and 15 males and 15 females were randomly selected per pen.

### Housing and Management PostPlacement

Chickens were housed in 27 floor pens of 2 m^2^ each in one room. Per treatment, there were 8 replicates and 1 spare pen to replace chickens that died or were culled in the first week. The replicates per treatment were randomly distributed within 8 blocks. Fresh wood shavings were used as bedding material and each pen contained 1 round hanging feeding pan and 1 drinking line with 7 nipples. Feed and water were provided ad libitum.

Chickens were fed a 3-phase diet, consisting of a crumbled starter diet (2,849 kcal ME/kg, 21.6% CP, 10.99 g/kg dLys) until 11 d of age, a pelleted grower diet between d 12 and 27 d of age (2,950 kcal ME/kg, 20.1% CP, 10.25 g/kg dLys, 3 mm pellet size), and a pelleted finisher diet between day 28 until d 38 of age (3,000 kcal ME/kg, 18.9% CP, 9.5 g/kg dLys, 3 mm pellet size; all produced by Research Diet Service, Wijk bij Duurstede, The Netherlands). Room temperature decreased from 34˚C at d 0 to 20°C at d 31 and remained at that level thereafter. Relative humidity was maintained between 50 and 60% between d 0 and 7 of age and between 40 and 70% between d 8 and 38 of age. During the first 3 d posthatch, a 23 h light and 1 hour darkness schedule was applied, and this schedule was gradually adjusted to 16 h of light and 8 h of darkness (10:00 PM until 6:00 AM) at d 9 of age.

All chickens received a vaccination with live Newcastle Disease (**NCD**) virus, strain C2 (Nobilis CD2, MSD Animal Health, Boxmeer, The Netherlands) by applying a droplet in the eye and the nostrils at day 0 and 14. Furthermore, all chickens were inoculated at d 28 with a live attenuated Infectious Bronchitis (**IB**) vaccine virus, serotype Massachusetts, strain Ma5 (Nobilis, IB M-A5, MSD Animal Health, Boxmeer, The Netherlands) by applying a droplet in the eye and the nostrils.

### Measurements

After transferring 18-d fertile eggs to the 3 different hatching systems, eggshell temperature (**EST**) of 6 eggs per treatment was measured every 10 min until the chickens emerged from the eggshell. EST sensors (NTC Thermistors: type DC 95, Thermometrics, Somerset, UK) were attached to the equator of 6 eggs by using heat conducting paste (Dow Corning 340 Heat Sink Compound, Dow Corning GmbH, Wiesbaden, Germany) and a permeable piece of tape of 2 by 2 cm. EST sensors were positioned at 3 locations within each hatching system (HF treatment: top, middle, bottom, HH and OH treatment: front, middle, back). Mean EST was calculated per treatment for the 10 min measurements and plotted against day of incubation.

At processing time that is standard for the different hatching systems (510 h after start of incubation for the HH and OH treatment, 516 h for the HF treatment), non-hatched eggs and second-grade chickens were removed and counted per basket (HH treatment), cradle (HF treatment), or pen (OH treatment). Unhatched eggs were opened and the stage of embryonic mortality (before or after d 18 of incubation) was determined as described by Lourens et al. (2006). The number of second grade chickens and hatch of transfer was calculated as a percentage of the alive embryos at d 18 of incubation per basket, cradle or pen.

At 24 h after placement in the research facility (d 1), chick quality characteristics were measured in 3 randomly chosen females and 3 randomly chosen males per pen. Chickens were weighed and chick length was measured by one person stretching the chicken along a ruler and measuring from the tip of the beak to the tip of the right middle toe excluding the nail (Hill, 2001). Navel condition was scored as 1 (good: closed and clean navel), 2 (moderate: black button up to 2 mm or black string) or 3 (poor: black button exceeding 2 mm or open navel area) (Molenaar et al., 2010). Red hocks and red beaks were scored as 0 (not present) or 1 (present) as described by Van den Brand et al. (2019).

Body weight was measured at d 0, 7, 14, 21, 28, and 35 of age. Daily mortality and culled chickens were recorded per pen. Total mortality for the first week and total production cycle mortality included chickens that died and that were culled and were expressed as a percentage of the total number of chickens per pen plus the number of replaced chickens.

At d 18 of age, blood samples were taken from the wing from 4 males and 4 females that were randomly selected per pen and collected in natrium heparinized tubes (Vacuette 4 mL FX, Greiner Bio-One). After centrifugation (10 min at 2,000×g for 10 min), plasma was decanted and stored at −20°C until analysis. NCD titers were analyzed with an ELISA kit (NDV Ab test, IDEXX, Hoofddorp, The Netherlands) as described in Wijnen et al. (2020).

At d 21 and 35 of age, 5 males and 5 females per pen were randomly selected to assess the following welfare indicators: footpad dermatitis, hock burn, cleanliness, skin lesions and gait score according to the Welfare Quality Assessment Protocol for Poultry (Welfare Quality, 2009). Scoring was performed by one single observer. Footpad dermatitis (**FPD**) was scored between 0 (no lesions) and 4 (ulcers or scabs, signs of hemorrhages, or deep dermatitis). Hock burns were scored between 0 (no lesions) and 4 (brown or black discoloration of the hock, total affected area >0.5 cm^2^). Cleanliness of plumage was scored between 0 (feathers and skin are totally clean) and 3 (feathers and/or skin of the belly is dirty). Skin lesions were scored between 0 (no lesions) and 2 (at least 1 lesion >2 cm diameter). Gait was scored between 0 (normal gait) and 5 (incapable of walking). On d 35, litter quality was scored between 0 (completely dry and loose) and 4 (very wet or completely capped with a crust) for all pens according to the Welfare Quality Assessment Protocol for Poultry (Welfare Quality, 2009).

At d 27 and d 31 (3 d after the IB vaccination), 2 male and 2 female chickens were randomly selected, weighed and killed. The trachea was dissected, and 2 trachea rings of approximately 0.5 cm were taken from each of the cranial, mid and caudal trachea. One ring was immediately snap frozen in liquid nitrogen and stored at −80°C for further processing for qPCR and the other immersed and fixed in 10% neutral-buffered formalin for histological analysis.

### RNA Isolation, cDNA Synthesis, Quantitative PCR (qPCR)

All three trachea specimens from d 31 were pooled and homogenized in Trizol and RNA was extracted and purified using the ZYMO Direct-zol RNA kit following manufacturer's instructions. Total RNA quantity and purity were determined by using the NanoDrop 260/280 system, accepting only samples with a ratio >2.0. For the quantification of cytokine and tight junction related mRNA, cDNA was synthesized with a QuantiTect reverse transcription kit (Qiagen). Real time PCR reactions were performed with SYBR green master mix (ThermoFisher Scientific, Waltham, MA) and measured in an Applied Biosystems 7500 system (ThermoFisher Scientific, Waltham, MA). Primers used for the gene expression analysis were extracted from literature and annotation is shown in Table 1. The 6 genes of interest were claudin, occludin (Gilani et al., 2018), nuclear factors Kappa B (NF-κB) (Chiang et al., 2009). IL8, interferon gamma (IFNγ) (Cornelissen et al., 2009), and IL10 (Genbank accession nr. EU999771, FP411-429, RP 498-477). Three house-hold genes were included in the analysis, that is, B2M (Gene ID 414830), RPL4 (Gene ID 415551), and SDHA (Gene ID 395758).

**Table 1:** Primers used for RT-qPCR.

Target	Sequence Forward primer	Sequence reverse primer
Housekeeping genes (REF)		
B2M	GCGGGCACCAAGAACGT	GTTGAAGGACATGTCGGAGTACTG
RPL4	TTATGCCATCTGTTCTGCC	GCGATTCCTCATCTTACCCT
SDHA	CAGGGATGTAGTGTCTCGT	GGGAATAGGCTCCTTAGTG
Genes of interest (GOI)		
Claudin	AAGGTGTACGACTCGCTGCT	CAGCAACAAACACACCAACC
Occludin	ACGGCAAAGCCAACATCTAC	ATCCGCCACGTTCTTCAC
IL8	ATTCAAGATGTGAAGCTGAC	AGGATCTGCAATTAACATGAGG
IL10	CGCTGTCACCGCTTCTTCA	TCCCGTTCTCATCCATCTTCTC
NFkB-1	GAAGGAATCGTACCGGGAACA	CTCAGAGGGCCTTGTGACAGTAA
IFNγ	TTCGATGTACTTGGAAATGC	TTGCATCTCCTCTGAGACTG

On each set of qPCR results a normalizer or calibrator was determined from all samples from the HH treatment (here used as reference group) and the average Ct from HH samples for each gene was calculated and used as calibrator. The DeltaCt for each gene was calculated by: Ct Calibrator - Ct Sample. To determine the relative quantity of expression of each gene/sample, the efficiency for each gene/primer based on slope given from the software was calculated and the relative quantity was calculated as: RQ = Efficiency ^ DeltaCt. To calculate the relative expression, the geometric mean of all housekeeping genes was calculated and the elative expression (**RE**) of each gene/sample was calculated as: RE = RQ GOI/RQ geomean HKs. In a following step, the average relative expression of the control group was calculated and used to determine the ratio for the HF and OH treatments and the fold change that was expressed as log2 fold change.

### Histology

All three tracheal rings of the chickens at d 27 and d 31 were embedded in paraffin wax and 4 µm tissue sections were processed routinely and stained with hematoxylin-eosin (H&E). Alterations in the tracheal mucosa were (semi)-quantitatively scored by determining the extent of epithelial damage, with score 0 = no epithelia degeneration, 1 = focal epithelial degeneration, 2 = multifocal epithelial degeneration and loss, 3 = diffuse epithelial loss, and the extend of inflammatory mucosal changes, with score 0 = no inflammatory cells, 1 = few inflammatory cells, 2 = influx of inflammatory cells, forming several layers and extending to less than 50% of the tracheal circumference or 3 = influx of inflammatory cells, forming several layers and extending to more than 50% of the tracheal circumference.

### Statistical Analysis

Data were analyzed within SAS (Version 9.4, SAS institute) and pen was the experimental unit for the analyses, unless stated differently. The following model was used:

$$Y_{i}=\mu +\text{hatching}\;\text{system}+e_{i}\;\left[1\right]$$

Hatch of transfer and second grade chicken were expressed as percentage of fertile eggs at d 18 of incubation per basket (HH treatment), cradle (HF treatment) or pen (OH treatment). Culled chicken and total mortality for wk 1 and total mortality were expressed as percentage of the total number of chickens per pen at placement plus the number of replaced chickens. Average weekly body weight per chicken was calculated by dividing chicken weight of the total pen by number of chickens present per pen. For all these parameters, a MIXED procedure was used with block as a random factor.

For body weight and length at d 1, a MIXED procedure was used and sexes and the interaction between treatment and sexes were added as fixed factors to model 1. NCD titers at d 18 were analyzed with MIXED procedure, using model 1 added with sexes and the interaction between treatment as fixed factors. Body weight at d 18 was added as a covariable. Log2 fold change data of the 6 genes of interest of male chickens were analyzed with a MIXED procedure, with body weight added as a covariable. For body weight and length at d 1 and NCD titers at d 18, chickens were measured individually and therefore pen (1–24) nested within block (1–8) was included as a random factor.

For navel condition at d 1, and welfare indicators at d 21 or d 35, a GLIMMIX procedure was used with model 1 added with sexes and the interactions with treatment as fixed factors. The multinomial cumulative logit function was used. For inflammation and epithelial scores at d 27 and 31, the same model was used and chicken weight at the respectively days was added as a covariable. For litter quality at d 35, a GLIMMIX procedure was used with model 1 and block (1–8) was added as a random factor. Because of the low prevalence of red beaks (n = 0) and red hocks (n = 2), and the absence of skin lesions on d 21, these characteristics were not statistically analyzed.

For all the measurements that were determined on individual chicken, pen (1–24) nested within block (1–8) was included as a random statement to the model. For all models that were used, nonsignificant interactions were removed from the models. Differences were considered significant at *P* ≤ 0.05 and least squares means were compared using Bonferroni adjustments. Unless stated differently, data are presented as Least squares means ± SEM in tables.

## RESULTS

### Hatch of Transfer and Eggshell Temperature

**Figure 1:**
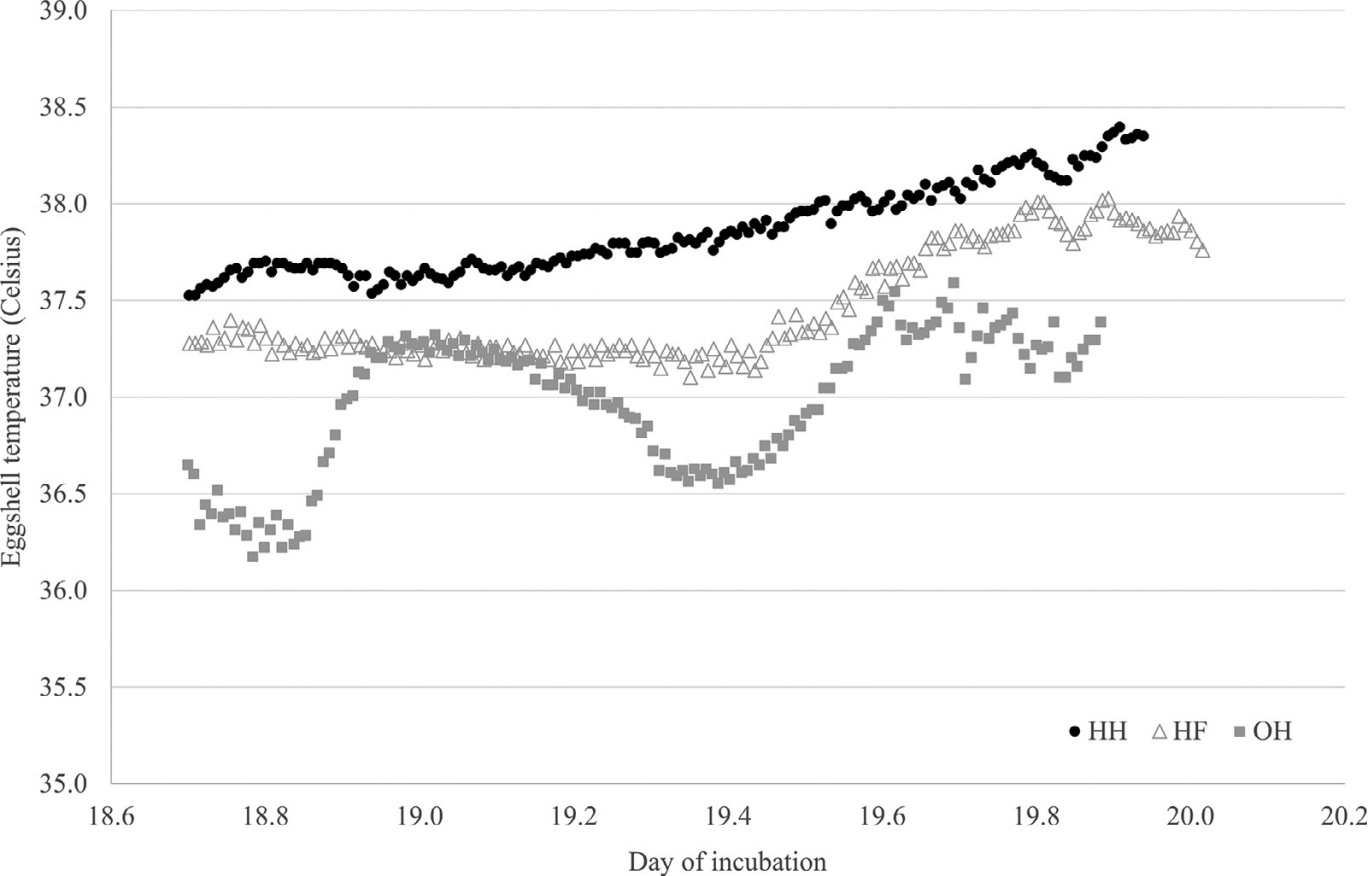
Mean eggshell temperature from day 18.7 until 20.0 of incubation of eggs that were hatchery-hatched (HH), hatchery-fed (HF), or on-farm (OH) hatched (n = 6 eggs per treatment group).

Hatch of transfer did not differ between the hatching systems and was 99.0% for the HH, 99.3% for the HF and 100% for the OH treatment (*P* = 0.20; SEM = 0.40). EST measurements per 10 min from d 18.7 until d 20 of incubation showed different patterns for the three hatching systems (Figure 1). In the HH treatment, EST increased from approximately 37.5°C until 38.4°C during the measuring period. In the HF treatment, EST was maintained around approximately 37.3°C between d 18.7 and 19.5°C of incubation and increased toward 38.0°C at day 19.8 of incubation. In the OH treatment, EST fluctuated between a minimum of 36.1°C (d 18.8 of incubation) and a maximum of 37.5°C (d 19.7 of incubation).

### Day-old Chick Quality Characteristics

Chick quality measurements at d 1 showed that HF chickens had the highest body weight, followed by OH chickens and HH chickens (*P* < 0.001; Table 2). Chick length at d 1 was longer for HF compared to the HH chickens, with OH chickens in between (*P* < 0.01). Navel condition did not differ between hatching systems (*P* = 0.37), but males had a worse navel condition score than females at day 1 (*P* = 0.045).

**Table 2:** Chick quality characteristics at day 1 of hatchery-hatched (HH), hatchery-fed (HF), or on-farm hatched (OH) broiler chickens.

Parameter*	n⁎⁎	HH	HF	OH	SEM	*P*-value treatment	*P*-value sex
Body weight (g)	8	46.6^c^	52.9^a^	50.4^b^	0.61	<0.001	0.19
Chick length (cm)	8	19.7^b^	20.0^a^	19.8^ab^	0.08	<0.01	0.94
Navel condition score	8	1.4	1.4	1.5	-	0.37	0.05#

⁎ Chick length was measured from the tip of beak to tip of the right middle toe, excluding the nail. Mean navel condition per treatment group, navel condition was scored as 1 (good), 2 (moderate), or 3 (poor).

⁎⁎ Number of pens per treatment; 3 randomly chosen males and females were measured per pen.

# Males had a lower navel condition score than females (1.5 vs. 1.3 respectively).

a-c LSmeans within a row lacking a common superscript differ (*P* ≤ 0.05).

### Performance during Grower Period

Body weight at d 0, measured at pen level, was highest in the HF treatment, followed by OH treatment and then the HH treatment (*P* < 0.001; Table 3). At d 7, 14, 21, 28, and 35, body weight of the HF and OH treatment was higher compared to the HH treatment (all *P* ≤ 0.01). Percentage of second grade chickens (1.6 ± 0.73% (mean ± SEM)), culled (1.1 ± 0.72%) and mortality of the first week (1.2 ± 0.74%) and total culled (1.3 ± 0.87%) and mortality from day 0 until day 38 (3.3 ± 1.10%) did not differ between treatments (all *P* ≥ 0.14).

**Table 3:** Body weight of hatchery-hatched (HH), hatchery-fed (HF), or on-farm hatched (OH) broiler chickens between day 0 and day 35 of age.

Day	n*	HH	HF	OH	SEM	*P*-value treatment
0	8	36.9^c^	42.5^a^	41.2^b^	0.38	<0.001
7	8	158.5^b^	181.4^a^	176.3^a^	1.60	<0.001
14	8	461.8^b^	497.8^a^	491.6^a^	4.25	<0.001
21	8	975.1^b^	1,022.3^a^	1,024.5^a^	7.58	<0.001
28	8	1,641.3^b^	1,694.2^a^	1,693.6^a^	14.32	<0.01
35	8	2,388.6^b^	2,454.7^a^	2,445.6^a^	16.10	0.01

⁎ Number of pens per treatment, starting with 15 males and 15 females per pen at day 0.

a-c LSmeans within a row lacking a common superscript differ (*P* ≤ 005).

### Welfare Indicators

**Figure 2:**
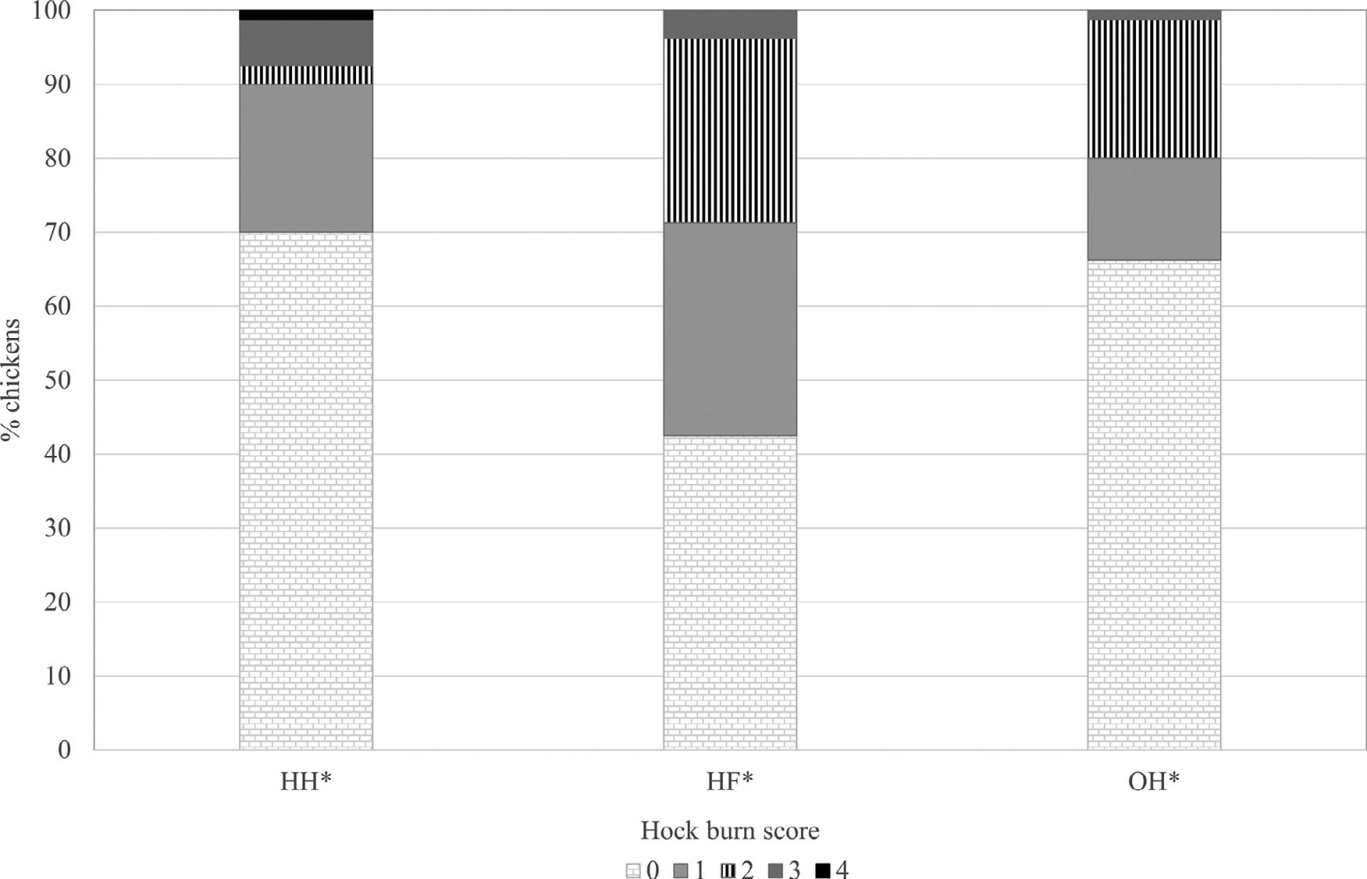
Percentage of chickens per hock burn score of male and male and female broiler chickens at day 35 of age that were hatchery-hatched (HH), hatchery-fed (HF), or on-farm (OH) hatched. *Hock burn was scored from 0 (no lesions) to 4 (brown or black discoloration of the hock, total affected area >0.5 cm^2^); 8 pens per treatment were sampled; 5 randomly chosen males and females were measured per pen. Hock burn score of HF chickens was significantly higher than HH and OH chickens (*P* = 0.01), no difference between males and females was found (*P* = 0.08).

Footpad dermatitis, hock burn, cleanliness, and gait score at d 21 did not differ between hatching systems or sexes (all *P* ≥ 0.16; Supplementary data). Footpad dermatitis, cleanliness, skin lesions and gait score at d 35 did not differ between hatching systems or sexes (all *P* ≥ 0.08; Supplementary data). Hock burn score was higher in HF than in HH and OH chickens (*P* = 0.01; Figure 2). The visual litter quality score at d 35 of age did not differ between hatching systems and was on average 3.8 (*P* = 0.40).

### Humoral Immune Response after NCD Vaccination

At d 18 of age, NCD antibody titres did not differ between treatments (*P* = 0.08) nor between sexes (*P* = 0.81) and was on average 2.2 ± 0.12.

### Tracheal Tissue Response to Attenuated IB Virus Infection

**Figure 3:**
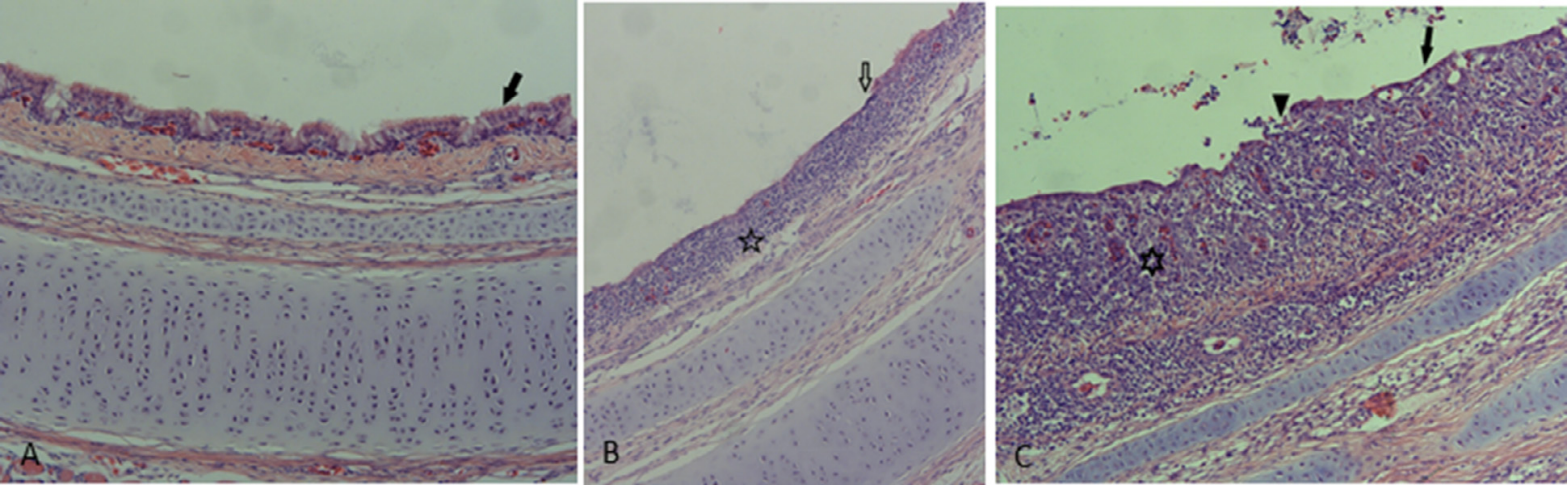
Trachea alterations at three days after IBV vaccination. (A) trachea without any changes and normal, ciliated epithelium (black arrow) (Chicken from HH treatment group); (B) mononuclear inflammatory cell infiltration in mucosa (open star) and focal epithelial degeneration and loss of cilia (open arrow) (Chicken from OH treatment group); (C) extended mononuclear infiltration in tracheal mucosa (open star) and epithelial degeneration and necrosis (arrowhead) (Chicken from HH treatment group). H&E staining, 20x objective magnification.

Pathological changes of the tracheal epithelium and inflammation of the tracheal mucosa at d 27 and 31 did not differ between hatching systems or sexes (*P* ≥ 0.23; Table 4). No inflammation of the tracheal mucosa was observed before infection at d 21 (Figure 3) (mean inflammation score 0.09, mean epithelial score 0). At d 31, 63% (OH-treatment group), 67% (HH-treatment group) and 77% (HF-treatment group) had inflammatory changes in 2 or 3 trachea rings, meaning that the inflammation was extended in the length of the trachea (descriptive statistics). Inflammation was characterized by focal to extended epithelial degeneration and necrosis accompanied by various degrees of lymphohistiocytic subepithelial inflammation. The mean inflammation score and epithelial damage score of all 3 trachea rings at d 31 was 1.15 and 0.63, respectively (Table 4).

**Table 4:** Histological changes in trachea of hatchery-hatched (HH), hatchery-fed (HF), or on-farm hatched (OH) broiler chickens before (day 27) and after (day 31) IBV vaccination.

Indicator	n⁎	HH	HF	OH	*P*-value treatment	*P*-value sex
Inflammation score⁎⁎						
Day 27	8	0.14 ± 0.42	0.05 ± 0.15	0.09 ± 0.19	0.62	0.63
Day 31	8	1.08 ± 0.89	1.29 ± 0.78	1.09 ± 0.69	0.64	0.24
Epithelial damage score#						
Day 27	8	0	0	0	-	
Day 31	8	0.50 ± 0.51	0.79 ± 0.53	0.61 ± 0.56	0.23	0.89

⁎ Number of pens per treatment, 2 randomly chosen males and females were measured per pen for inflammation and epithelial score, 3 trachea locations (top, middle, bottom) were scored.

⁎⁎ Inflammation was scored as 0 = no inflammatory changes, 1 = few inflammatory cells, 2 = moderate influx of inflammatory cells, forming several layers and extending to less than 50% of the tracheal circumference or 3 = moderate influx of inflammatory cells, forming several layers and extending to more than 50% of the tracheal circumference, table contains means ± SD of 3 trachea rings.

# Epithelial damage was scored as 0 = no change, 1 = focal epithelial degeneration, 2 = multifocal epithelial degeneration and loss, 3 = diffuse epithelial loss or the extend of inflammatory mucosal changes, table contains means ± SD of 3 trachea rings.

**Figure 4:**
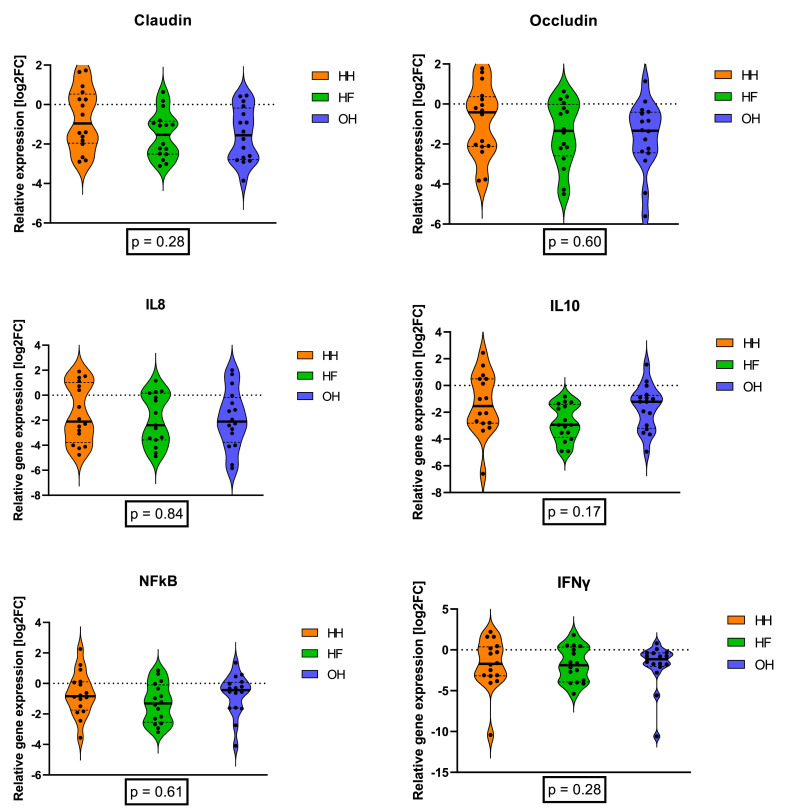
Log2 fold change of genes in the trachea related to inflammation and epithelia integrity of male chickens that were hatchery-hatched (HH), hatchery-fed (HF), or on-farm hatched (OH). Fold changes were calculated in relation to the mean expression level of the HH-treatment group. Eight pens per treatment and 2 randomly chosen males per pen were sampled.

In the trachea tissue homogenate, the relative mRNA expression of the 6 genes of interest: claudin, occludin, IL8 and IL10, nuclear factors Kappa B (NF-κB), and interferon gamma (IFNγ) did not differ between treatments (all *P* ≥0.17; Figure 4).

## DISCUSSION

In this study, three hatching systems were compared regarding effects on chick quality, welfare and health of broiler chickens. Body weight at d 1 was approximately 10% higher for HF and OH compared to HH chickens, which has also been found in other studies about alternative hatching systems (de Jong et al., 2019; de Jong et al., 2020; Souza da Silva et al., 2021). The major contribution to this increase in body weight was probably the immediate posthatch feed and water provision (Gonzales et al., 2003), that was around 30 h earlier for HF and OH compared to HH treatment group in this study. Early provision of feed and water has been shown to stimulate intestinal development of especially the jejunum and ileum and the increased digestion and absorption capacity of these organs seem to enhance nutrient assimilation and body development (Noy and Sklan, 1998; Geyra et al., 2001). Within this study, body weight of HF chickens was 2.5 g (+4.7%) higher than OH chickens which was possibly the result of a higher feed intake and/or different diet composition of the prestarter.

No difference in navel condition and/or red hocks and beaks was found between treatment groups at d 1 and this differed from other studies where OH chickens were often found to be worse compared to HH and HF chickens (van de Ven et al., 2012; de Jong et al., 2019; de Jong et al., 2020; Jessen et al., 2021b). Poor navel conditions and red hocks can be related to the temperature that the embryos experienced during the final days of incubation (Wilson, 2004). High embryo temperatures, expressed in an EST above 38.9°C, have been found to result in poor navel conditions, red hocks and red beaks as a result of more difficulties to emerge from the eggshell (Leksrisompong et al., 2007; Molenaar et al., 2010). A continuous EST between 37.5°C and 38.0°C throughout incubation has been shown to result in the highest hatchability and chicken development at hatch (Lourens et al., 2005; Leksrisompong et al., 2007; Wijnen et al., 2020). In the current study, EST was monitored from d 18.7 until 20.0 of incubation in all hatching systems. None of the treatments had an EST >38.9°C, which probably explains the lack of differences between treatment groups for navel condition scores, red hocks, and red beaks.

Larger fluctuations and lower EST were found in the OH compared to HH and HF treatment in this study and are probably related to differences in system design and temperature settings. The decrease to 36.5°C around d 19.2 of incubation in the OH treatment was the result of colder outside temperatures at night. EST that was used as a reference for embryo temperature is the result of the metabolic heat production of the embryo, air temperature and heat loss from the egg to the surrounding environment (Meijerhof and van Beek, 1993). Assuming that heat production was similar between eggs within the different hatching systems, eggshell temperature was mainly influenced by air temperature and heat loss capacity. Air temperature was set lower in the OH compared to HH and HF treatment to increase the heat loss capacity due to the lower air velocity in the OH compared to the HH and HF treatment.

Body weight of the OH and HF treatment was higher compared to the HH treatment throughout the growout period, as was also found by Souza da Silva et al. (2021). In the study of Souza da Silva et al. (2021) and the present study, chickens of a young broiler breeder flock were used and their performance seems to be influenced for a longer period of time by alternative hatching systems providing immediately feed and water than the performance of chickens from prime and old breeder flocks (van de Ven et al., 2011; Hollemans et al., 2018; Souza da Silva et al., 2021). This may be related to the smaller egg and chicken size and lower thermoregulatory capacity of chickens of young compared to older breeder flocks (Weytjens et al., 1999) as a result of the lower energy deposition throughout incubation (Nangsuay et al., 2013). These aspects may interact with early feeding as well as a higher sensitivity to early life stressors (Peixoto et al., 2021) and resulted in a positive effect on growth performance in the current study. No effect of second grade chickens, or cull and mortality rates were found, which is comparable with earlier studies (van de Ven et al., 2011; de Jong et al., 2019; Souza da Silva et al., 2021).

Except for hock burn at d 35, no differences in welfare indicators were found for the 3 treatment groups. Other studies in (semi)-commercial conditions found a lower or tendency for a lower footpad dermatitis (**FPD**) in chickens hatched in alternative hatching systems proving feed and water immediately after hatch compared to conventional hatching systems with a delay in feed and water provision (de Jong et al., 2019; Giersberg et al., 2021). It was suggested that the improved FPD was related to a better litter quality because of a lower moisture content (de Jong et al., 2014; de Jong et al., 2019). In the current study, chickens were housed in small pens which resulted in poor litter conditions at the end of the production cycle in all treatment groups. This possibly explained the lack of difference in FPD incidence and/or severity. The higher hock burn score in the HF compared to HH and OH treatment was not found in other studies (de Jong et al., 2019; Giersberg et al., 2021; Jessen et al., 2021b). It can be hypothesized that chickens of the HF treatment showed more sitting behavior and were longer in contact with the litter. However, this was not confirmed by the other welfare measurements performed or the activity measurements that were assessed throughout the present study in a separate room by a RFID system (Giersberg et al., unpublished results) or another study of Giersberg et al. (2020) assessing general behavior of HH and OH broiler chickens, although no HF treatment was included in this study.

Previous studies have suggested that provision of early feeding can affect immune system development and elicit enhanced humoral immune responses (Ben Nathan et al., 1977; Dibner et al., 1998; Bar Shira et al., 2005; Panda et al., 2015; Hollemans et al., 2021). To examine this in the alternative hatching systems, chickens were vaccinated twice with a commercially available NCD vaccine. However, no difference in NCD titers was observed between the hatching systems. This seems to be in accordance with earlier findings that the humoral immune response is not enhanced by early feeding (Simon et al., 2015; Hollemans et al., 2021; Ivarsson et al., 2022).

Disease resilience, that is, the ability to cope with an infection (Doeschl-Wilson et al., 2021), can contribute to a reduction of clinical signs in the presence of an infection. Disease resilience can genetically be improved by breeding, but also by means of animal management like enriched housing (van Dixhoorn et al., 2016; Parois et al., 2022). To study the effects of animal management on disease resilience under experimental conditions often subclinical infection models are used in chickens, like a subclinical necrotic enteritis (**NE**) model (Wijnen et al., 2021) or a coccidia infection model (Santos et al., 2022). Wijnen et al. (2021) found a tendency for a lower total mortality after a NE challenge in early fed compared to delayed fed (51–54 h) broiler chickens, but no differences were found in body weight changes or several morbidity parameters after the NE challenge.

Respiratory infections are common in chickens and can be caused by viruses and bacteria and can lead to respiratory diseases of the upper or lower respiratory tract. Infectious bronchitis virus (**IBV**) is a highly contagious infection in chicken, which replicates in various parts of the respiratory tract, including the trachea and can induce severe respiratory disease often in combination with bacterial infections. Vaccination against IBV is commonly applied in practice, mostly with attenuated live IBV vaccine viruses. This can elicit innate and adaptive immune responses at mucosal surfaces, but also lead to inflammation of the tracheal mucosa (Chhabra et al., 2015; van der Eijk et al., 2022) and epithelial damage, which is to a defined extend an accepted effect of such vaccines as described in the European Pharmocopoeia (Ph. Eur 7.7 0442 (04/2013)). To study effects of hatching systems on disease resilience, and in particular reduced susceptibility to develop pathological changes in the respiratory tract, an infection with a live-attenuated IBV vaccine virus was applied in the current study. As expected, the infection did not lead to clinical disease symptoms, but clear epithelial and inflammatory changes were observed in all treatment groups. However, no differences were observed between groups in the extent or severity of inflammation of the trachea. To further characterize and quantify epithelial damage and inflammation, transcriptional expressions of genes related to epithelial integrity or inflammatory response were examined. Transcriptional studies on tracheal epithelium after IBV infections have shown effects amongst others on pathways concerning signaling processes relevant for adherence junctions and inflammatory pathways (Hashemi et al., 2020). However, in comparison with the HH treatment, that acted as control group, no differences in gene expression were found at three days after infection in the HF and OH treatment groups. The selected genes of interest related to epithelial integrity were the two most important components of the tight junction proteins (**TJP**): claudin and occludin. They play a role in the permeability of epithelial cells (Gilani et al., 2018) and control the flow of molecules between cells. The genes assessed in the current study related to inflammation; IFN-y, Il-8, Il-10, are cytokines that are involved in the innate and adaptive immune response and control the response of T-cells after an infection (Rebel et al., 2005). IFNγ can be produced by T helper 1 (Th1) cells and increases the cell-mediated response during an infection, but can be counteracted by IL-10 that has an anti-inflammatory and immune suppressive effect (Cornelissen et al., 2009). NF-κB has a central role in the innate and adaptive immune response toward an infection (Chiang et al., 2009; Oeckinghaus and Ghosh, 2009) and activates, amongst others, the production of the cytokine IL-8 (Liu et al., 2017). The lack of difference in transcriptional expression of the selected genes between the 3 hatching systems indicates that with this infection model, the early immune response seems not to be influenced by hatching systems. However, the extent of inflammatory changes after using a vaccine virus was surprisingly high and an increased risk of secondary infections of the respiratory tract cannot be excluded. In a recent study, this infection model was also used to study differences in disease susceptibility after chronic exposure to endotoxins, but no differences in respiratory disease manifestation were observed in the respiratory tract (van der Eijk et al., 2022). More refined transcriptional profiling techniques like RNA-seq might be necessary to elucidate more precisely differences in responses to IBV vaccination between different hatching systems. This might be especially important information in relation to commercial farm conditions, where sanitary conditions are often less controlled as compared to research facilities.

In conclusion, in controlled experimental conditions, alternative hatching systems that included provision of feed and water at the hatchery (HF) or on-farm (OH) resulted in better chick quality in terms of body weight and chick length and posthatch body weight gain but showed only one effect on selected chicken welfare and health parameters. No effect of hatching system on humoral immune response or disease resilience was found.

## Appendix Supplementary materials

Image, application 1
