# Supplementary material for: Effects of hatching system on chick quality, welfare and health of young breeder flock offspring
Source: Poult Sci. 2022 Dec 21;102(3):102448. doi: 10.1016/j.psj.2022.102448 (PMC9846018; doi:10.1016/j.psj.2022.102448)
Supplement: Supplementary file 1 [file mmc1.docx]

**Supplementary data**

Table 1: Percentage of chickens per score for various animal-based welfare indicators of male and female broiler chickens at day 21 of age that were hatchery-hatched (HH), hatchery-fed (HF), or on-farm (OH) hatched.

| Score* | Food pad dermatitis | | | Hock burn | | | Cleanliness | | | Gait score | | |
| --- | --- | --- | --- | --- | --- | --- | --- | --- | --- | --- | --- | --- |
|  | HH | HF | OH | HH | HF | OH | HH | HF | OH | HH | HF | OH |
| 0 | 75 | 71 | 68 | 99 | 98 | 95 | 34 | 39 | 54 | 0 | 0 | 0 |
| 1 | 15 | 24 | 24 | 1 | 3 | 4 | 66 | 61 | 46 | 5 | 3 | 6 |
| 2 | 10 | 5 | 9 | 0 | 0 | 1 | 0 | 0 | 0 | 86 | 90 | 89 |
| 3 | 0 | 0 | 0 | 0 | 0 | 0 | 0 | 0 | 0 | 9 | 8 | 5 |
| 4 | 0 | 0 | 0 | 0 | 0 | 0 |  |  |  | 0 | 0 | 0 |
| 5 |  |  |  |  |  |  |  |  |  | 0 | 0 | 0 |
| P-value** |  | | |  | | |  | | |  | | |
| Treatment | 0.79 | | | 0.16 | | | 0.16 | | | 0.48 | | |
| Sex | 0.64 | | | 0.69 | | | 0.69 | | | 0.25 | | |

*Footpad dermatitis was scored from 0 (no lesions) to 4 (ulcers or scabs, signs of haemorrhages, or deep dermatitis), hock burns was scored from 0 (no lesions) to 4 (brown or black discoloration of the hock, total affected area>0.5 cm^2^), cleanliness was scored from 0 (feathers and skin are totally clean) to 3 (feathers and/or skin of the belly is dirty), gait was scored from 0 (normal gait) to 5 (incapable of walking), skin lesions were not present, and data was not analysed; **8 pens per treatment were sampled; 5 randomly chosen males and females were measured per pen

Table 2: Percentage of chickens per score for various animal-based welfare indicators of male and female broiler chickens at day 35 of age that were hatchery-hatched (HH), hatchery-fed (HF), or on-farm (OH) hatched.

| Score* | Food pad dermatitis | | | Cleanliness | | | Skin lesions | | | Gait score | | |
| --- | --- | --- | --- | --- | --- | --- | --- | --- | --- | --- | --- | --- |
|  | HH | HF | OH | HH | HF | OH | HH | HF | OH | HH | HF | OH |
| 0 | 15 | 14 | 18 | 0 | 0 | 0 | 82 | 74 | 88 | 0 | 0 | 0 |
| 1 | 19 | 16 | 28 | 64 | 1 | 4 | 16 | 21 | 10 | 1 | 0 | 0 |
| 2 | 65 | 70 | 54 | 36 | 53 | 56 | 1 | 5 | 3 | 31 | 25 | 34 |
| 3 | 1 | 0 | 1 | 0 | 46 | 40 |  |  |  | 66 | 75 | 66 |
| 4 | 0 | 0 | 0 |  |  |  |  |  |  | 1 | 0 | 0 |
| 5 |  |  |  |  |  |  |  |  |  | 0 | 0 | 0 |
| P-value** |  | | |  | | |  | | |  | | |
| Treatment | 0.22 | | | 0.67 | | | 0.11 | | | 0.11 | | |
| Sex | 0.08 | | | 0.97 | | | 0.13 | | | 0.13 | | |

*Footpad dermatitis was scored from 0 (no lesions) to 4 (ulcers or scabs, signs of haemorrhages, or deep dermatitis), cleanliness was scored from 0 (feathers and skin are totally clean) to 3 (feathers and/or skin of the belly is dirty), skin lesions were scored from 0 (no lesions) to 2 (at least 1 lesion >2 cm diameter), gait was scored from 0 (normal gait) to 5 (incapable of walking), **8 pens per treatment were sampled; 5 randomly chosen males and females were measured per pen
